# Development of a peer support intervention to encourage dietary behaviour change towards a Mediterranean diet in adults at high cardiovascular risk

**DOI:** 10.1186/s12889-018-6108-z

**Published:** 2018-10-22

**Authors:** Claire T. McEvoy, Sarah E. Moore, Katherine M. Appleton, Margaret E. Cupples, Christina Erwin, Frank Kee, Lindsay Prior, Ian S. Young, Michelle C. McKinley, Jayne V. Woodside

**Affiliations:** 1 Centre for Public Health, Queen’s University Belfast, Grosvenor Road, Belfast, BT12 6BJ UK; 2 Research Centre for Behaviour Change, Department of Psychology, Faculty of Science and Technology, Bournemouth University, Fern Barrow, Talbot Campus, Bournemouth, BH12 5BB UK; 3 UK Clinical Research Collaboration, Centre of Excellence for Public Health, Queens University Belfast, Grosvenor Road, Belfast, BT12 6BJ UK

**Keywords:** Mediterranean diet, peer support, behaviour change wheel, interventiondevelopment

## Abstract

**Background:**

Mediterranean diet (MD) interventions are demonstrated to significantly reduce cardiovascular disease (CVD) risk but are typically resource intensive and delivered by health professionals. There is considerable interest to develop interventions that target sustained dietary behaviour change and that are feasible to scale-up for wider public health benefit. The aim of this paper is to describe the process used to develop a peer support intervention to encourage dietary behaviour change towards a MD in non-Mediterranean adults at high CVD risk.

**Methods:**

The Medical Research Council (MRC) and Behaviour Change Wheel (BCW) frameworks and the COM-B (Capability, Opportunity, Motivation, Behaviour) theoretical model were used to guide the intervention development process. We used a combination of evidence synthesis and qualitative research with the target population, health professionals, and community health personnel to develop the intervention over three main stages: (1) we identified the evidence base and selected dietary behaviours that needed to change, (2) we developed a theoretical basis for how the intervention might encourage behaviour change towards a MD and selected intervention functions that could drive the desired MD behaviour change, and (3) we defined the intervention content and modelled outcomes.

**Results:**

A theory-based, culturally tailored, peer support intervention was developed to specifically target behaviour change towards a MD in the target population. The intervention was a group-based program delivered by trained peer volunteers over 12-months, and incorporated strategies to enhance social support, self-efficacy, problem-solving, knowledge, and attitudes to address identified barriers to adopting a MD from the COM-B analysis.

**Conclusions:**

The MRC and BCW frameworks provided a systematic and complementary process for development of a theory-based peer support intervention to encourage dietary behaviour change towards a MD in non-Mediterranean adults at high CVD risk. The next step is to evaluate feasibility, acceptability, and diet behaviour change outcomes in response to the peer support intervention (change towards a MD and nutrient biomarkers) using a randomized controlled trial design.

## Background

Cardiovascular disease (CVD) and type 2 diabetes (T2DM) are major public health concerns. Risk of these diseases can be significantly reduced by modifying lifestyle behaviours, such as diet. The Mediterranean diet (MD), rich in fruit, vegetables, wholegrain, nuts, olive oil and oily fish, low in processed foods and moderate in alcohol intake, is rated as the most likely dietary pattern to protect against coronary disease [1] and has been demonstrated, in a randomised controlled trial (RCT) setting, to significantly reduce the risk of developing CVD [2] and T2DM [3]. However, previous interventions to encourage MD behaviour change have used resource intensive methods [2, 3] which may be challenging for some healthcare systems to roll out to an ‘at risk’ or general population. There is a need to understand how to support dietary behaviour change toward a MD, particularly in non-Mediterranean population (adults living in a non-Mediterranean country), using approaches that are cost-effective, practical and feasible to implement for public health.

Peer support, defined as: ‘the provision of emotional, appraisal, and informational assistance by a created social network member who possesses experiential knowledge of a specific behaviour or stressor and similar characteristics as the target population, to address a health-related issue’ [4], may offer an alternative method of encouraging dietary change. One RCT, to date, has focused on a peer support behaviour change intervention promoting the MD. A six-month trial of the Mediterranean Lifestyle Programme demonstrated significant improvements in dietary behaviour and glycaemic control in postmenopausal women with existing diabetes [5]. This intervention targeted several health behaviours, including diet, physical activity, smoking and stress, and was delivered using a combination of peer and health professional support strategies. To our knowledge, there are no RCTs examining the effectiveness of exclusive peer-led support on adoption of a MD.

Dietary behaviour is complex and influenced by many factors interacting at psychological, social and environmental levels [6, 7]. Complex interventions aimed at changing behaviour often contain a number of components that can act independently and inter-dependently [8]. To date, there is limited evidence of the effectiveness of dietary interventions for sustained behaviour change [9] which may, in part, be attributable to inadequate intervention design [10]. There is a need to better understand how dietary interventions work, for whom, and in what context, to enable reproducibility of interventions and allow for effective translation of research into public health policy.

Designing a dietary behaviour intervention is a process that requires planning [11]. The most recent Medical Research Council (MRC) framework [11] provides guidance for the systematic development and testing of complex health interventions. A phased iterative approach consisting of development, feasibility and piloting, evaluation and implementation, is recommended, and has been applied in the development of complex health interventions across a wide variety of populations and settings [12–16]. The MRC framework advises that intervention design should be based on a theoretical understanding of how an intervention causes behaviour change. This is important as evaluation of theory-based interventions can elucidate reasons why interventions succeed or fail and how they might be optimised for specific populations. However, there is no consensus on the best method(s) to incorporate theory into intervention design.

Numerous frameworks of individual and population behaviour change are available to address the complexities involved in theory-based intervention development, examples include Intervention Mapping [17], Precede-Proceed Planning model [18] and the Behaviour Change Wheel (BCW) [19]. The BCW evolved from a synthesis of 19 behaviour change frameworks and provides a comprehensive approach to aid behaviour change intervention design. The BCW framework is underpinned by the Capability, Opportunity, Motivation, Behaviour (COM-B) model, which argues that for any behaviour to occur there must be: (i) *capability* to perform the behaviour (people must have the physical or psychological strength to perform the behaviour; e.g., sufficient knowledge and skills), (ii) *opportunity* for the behaviour to occur (people must have a conducive physical and social environment; e.g. affordable, accessible and socially/culturally acceptable) and, (iii) *motivation* to do the behaviour (people must have strong motivation which can be reflective (e.g. conscious planning or beliefs about what is good or bad) and/or automatic; (e.g. emotional reactions and reflex responses) [20]. According to this model, successful dietary behaviour change towards a MD in the population will involve changing one or more of these interacting components. Applying COM-B to the early stages of intervention development can help to identify which components need to change in order for the behaviour to occur in the population [20].

This paper summarises the process used to develop a theory-based, culturally-tailored peer support intervention to encourage dietary behaviour change toward a MD. The development process was guided by the MRC and BCW frameworks. The target population for intervention is Non-Mediterranean individuals at high risk of developing a primary CVD event, as greater MD adherence has been previously shown to significantly reduce CVD risk in a similarly high risk Mediterranean population [2].

## Methods

**Fig. 1:**
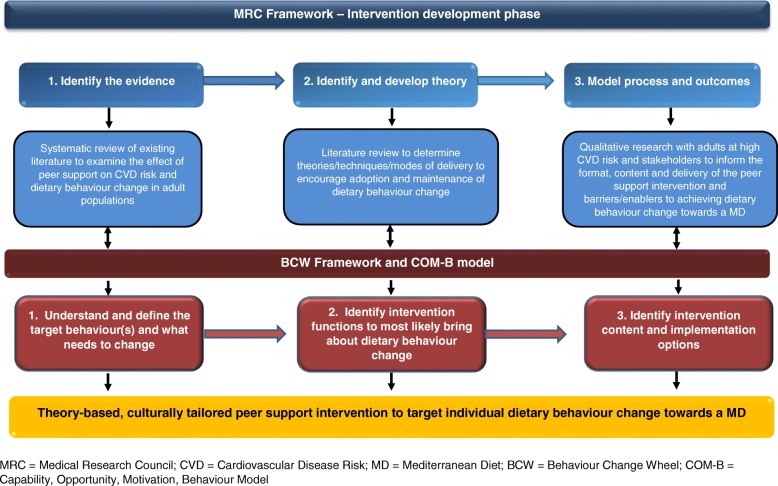
Process to develop a peer support intervention for dietary behaviour change toward a Mediterranean diet

The process used to develop a theory-based, culturally tailored peer support intervention to encourage MD dietary behaviour change is shown in Figure 1. The MRC framework was used as the overarching guide, while the BCW and COM-B model was applied to define the target behaviours and select the most suitable intervention components (functions and techniques) and implementation approach, based on existing literature and formative work with the target population. Ethical approval for the study was granted by the Health and Social Care, Office for Research Ethics Committees Northern Ireland (reference: 12/NI/0043).

There were 3 stages in the peer support intervention development process. The methods used in each stage of the applied MRC and BCW frameworks are described in more detail below.

### Stage 1

#### MRC Stage 1: Identifying the evidence base

In this first stage, we reviewed existing literature to identify similar peer support interventions and provide data on outcome and process evaluation methods to inform the development of a peer support intervention to encourage dietary behaviour change in the target population, (see Figure 1).

##### BCW Stage 1: Understanding and selecting target dietary behaviours

To change dietary behaviour there is a need to understand why behaviours are as they are and what needs to shift for the desired behaviour to occur [21]. Therefore, we reviewed evidence from other RCTs examining MD behaviour change [2, 22] to define MD behaviours and to inform the primary outcome assessment of MD behaviour change. In addition, the COM-B model was used to understand dietary behaviour in the context in which it occurs in our target population. We conducted focus group discussions (*n* = 12) with 67 adults (27 male) at high CVD risk, to explore the barriers and enablers to capability, opportunity, and motivation for enacting MD behaviour in a ‘real life’ individual context. Following iterative focus group analysis, further structured interviews with both the target population (*n*=19) and a combination of health professionals, community health workers and charity organisation personnel (*n* = 15) were conducted to gain a deeper understanding of the contextual factors influencing MD behaviour change. We then conducted a COM-B analysis of the qualitative data to determine which theoretical domains need to change for adoption of MD behaviour to occur in the target population.

### Stage 2

#### MRC Stage 2: Developing a theoretical basis for the intervention

Incorporating theory in the design of behaviour change interventions has been shown to improve effectiveness and can help to elucidate causal pathways of how an intervention works to change health behaviour [11]. In stage 2 of intervention development, we identified important psychological theories from existing literature to gain insight into the process of how peer support is likely to change individual dietary behaviour.

##### BCW Stage 2: Identifying intervention functions most likely to bring about behaviour change towards a MD in the target population

The COM-B analysis conducted in *BCW Stage 1 above* was linked to specific BCW intervention functions to identify strategies most likely to be effective to facilitate change MD behaviour change in a peer support intervention. The nine BCW intervention functions to choose from are: education, persuasion, incentivisation, coercion, training, restriction, environmental restructuring, modelling, and enablement [20].

### Stage 3

#### MRC Stage 3: Modelling processes and outcomes

In this stage, literature reviews and the views of our target population during stage 1 were used to tailor the peer support intervention appropriately. The focus group discussions with the target population outlined above were also used to determine the preferred peer support mode(s) of delivery. Further individual user preferences (individual ranking of peer support modes of delivery and ratings for peer supporter characteristics) were quantitatively recorded following each focus group discussion.

##### BCW Stage 3: Identifying intervention content and implementation options

BCW Stage 3 aimed to identify the peer support intervention content in terms of BCTs that would best deliver the identified intervention functions and drive the change in MD behaviour in the population. BCTs are defined as the ‘active ingredients’ in an intervention designed to bring about change [21], and examples include goal setting, self-monitoring of behaviour and social support. Two trained coders used the Behaviour Change Technique Taxonomy v1 (BCTTv1) [23] and the diet-specific behaviour taxonomy (CALO-RE) [24], to identify the most likely BCTs to effectively deliver the identified intervention functions within the peer support intervention. Identified BCTs were mapped to theoretical domains identified via the COM-B analysis and literature review.

The overall findings from the tasks performed under the MRC and BCW frameworks were synthesised to design the peer support intervention to encourage dietary behaviour change towards a MD in adults at high CVD risk.

## Results

### Stage 1

#### MRC Stage 1: Identifying the evidence base

Evidence synthesised from literature reviews are discussed in context under the results sections below. In addition, two systematic reviews of published intervention studies were conducted by the research team to examine the effect of peer support on (i) CVD risk (PROSPERO 2014:CRD42014006291) and, (ii) dietary behaviour change (PROSPERO 2014:CRD42014009994) to address gaps in the evidence base. Abstracts of systematic reviews have been submitted and are currently in press.

##### BCW Stage 1: Understanding and selecting target dietary behaviours

From reports of previous RCTs, the MD was identified as the dietary pattern with the strongest evidence base for CVD prevention [2] and is characterised by a high intake of fruit, vegetables, wholegrains, legumes, nuts and extra-virgin olive oil; a moderate intake of fish and poultry; a low intake of red meat, processed meat and confectionary; and moderate alcohol intake [2]. Hence, there are a range of nutritional behaviours that constitute a MD [25]. Individual MD adherence is determined using a Mediterranean Diet Score (MDS) [2, 22], where the frequency of consumption for a given portion of each specified food component is reported, and MDS is calculated as the sum score for included food components. Several variations of MDS systems are available in different populations, with each MDS comprising a similar ordinal scale (mostly ranging from 9 to 18). A recent meta-analysis reported that a 2-point increase in MDS was associated with a 10% reduction in CVD incidence and mortality [26]. Furthermore, this degree of MDS increase has been shown to be feasible in a non-Mediterranean population [27]. An evaluation of MD education, delivered by a dietitian, reported a significant increase in MDS (approximately 2-3 points) over 6 months in Northern Irish adults with low baseline MD adherence and pre-existing CVD [28]. Therefore, target behaviour change for the peer support intervention was defined as a ≥ 3 point increase in MDS from baseline to 6 months (adoption) as this change is likely to be both feasible and clinically important. The dietary behaviours to achieve increase in MDS were based on data from previous MD interventions [2, 22] and culturally adapted to incorporate specific food preferences of a non-Mediterranean population as shown in Table 1.

**Table 1:** Key diet behaviours to assess change towards a Mediterranean diet

Dietary behaviours	Targets to determine MD change	
	Daily	Weekly
*Increase* Monounsaturated fat (MUFA)	Olive/rapeseed oils ≥ 4 tblsp (60mls); MUFA-rich spreads ≥ 3 tsp (15g)	Natural nuts ≥ 3 handfuls (90g)
*Increase fruit and vegetables*	Fruits ≥ 2 portions (160g) Vegetables ≥ 3 portions (240g)	Legumes ≥ 3 portions (240g)
*Increase wholegrains*	Preferential consumption of wholegrain cereals over white varieties	
*Increase fish (particularly oily fish)*		Fish ≥ 3 servings (420g) (not battered or crumbed)
*Reduce meat*		Red meat ≤ 2 servings (300g) Processed meat ≤ 1 serving (150g)
*Reduce confectionary*		Confectionary ≤ 3 servings
*Moderate alcohol*		Alcohol (if consumed) 125-375 ml ≥ 3 days

*MDS* Mediterranean Diet Score

Findings from our focus groups and interviews in relation to barriers for adoption of a MD are reported in full elsewhere [28]. This qualitative work was used to inform the COM-B analysis which identified barriers in capability, opportunity and motivation for achieving dietary change toward a MD in our target population as shown in Table 2.

**Table 2:** COM-B analysis demonstrating intervention functions and BCTs to change diet in target population

Barriers to adopting a MD in adults at high CVD risk [28]	COM-B analysis	Intervention functions	BCTs [23] to best serve intervention functions
Lack of knowledge about the types/proportions of foods consumed	Psychological capability	Education Training Enablement	Instruction on how to perform the behaviour Demonstration of the behaviour
Limited cooking skills to prepare meals	Physical capability	Training Enablement	Instruction on how to perform the behaviour Behavioural practice Demonstration of the behaviour
Resistance to change eating habits established since childhood, especially to reduce intake of red/processed meats and sweet foods	Automatic motivation	Modelling Persuasion Enablement	Social support (unspecified) Problem-solving Goal setting (behaviour) Action planning Feedback on behaviour Set graded tasks
Increased cost for purchasing key foods especially olive oil, nuts and fruits and vegetables	Physical opportunity	Training Enablement	Instruction on how to perform the behaviour Demonstration of the behaviour
Increased time to prepare meals owing to busy lifestyles	Physical opportunity	Training Enablement	Instruction on how to perform the behaviour Demonstration of the behaviour
Limited availability of fresh foods especially fresh fish, fruit and vegetables	Physical opportunity	Training Enablement	Instruction on how to perform the behaviour
Lack of understanding about the health benefits of adopting a MD	Psychological capability	Education Training Enablement	Information about health consequences Biofeedback
Negative attitude toward increasing total fat intake	Reflective motivation	Education Persuasion	Information about health consequences Instruction on how to perform the behaviour Demonstration of the behaviour
Cold climate making it difficult to eat foods such as salads, fruit and vegetables	Physical opportunity	Training Enablement	Social support (unspecified) Verbal persuasion Problem-solving
Cultural norms making it difficult to change dietary behaviour	Social opportunity	Modelling Enablement	Instruction on how to perform the behaviour Demonstration of the behaviour
Negative attitude toward the taste of key foods especially olive oil, nuts and fish	Reflective motivation	Education Persuasion	Instruction on how to perform the behaviour Demonstration of the behaviour Social support (unspecified)

*MD* Mediterranean diet, *CVD* Cardiovascular, *COM-B* Capability, Opportunity, Motivation – Behaviour Model, *BCTs* Behaviour Change Techniques

### Stage 2

#### MRC framework Stage 2: Developing a theoretical basis for the intervention

Peer support is underpinned by the social support theory defined as ‘the process through which social relationships might promote health and well-being’ [29]. However, the processes by which peer support can influence health behaviours and outcomes are not fully known and may incorporate two main hypotheses. Firstly, the ‘Direct Effect hypothesis’ postulates that peer support could reduce feelings of isolation and loneliness, provide information about benefits of behaviours, such as diet, that positively improve health and well-being and encourage adoption and maintenance of new behaviours [4]. Secondly, the ‘Buffering Effect hypothesis’ suggests that peer support can help people to engage in problem solving and develop and strengthen coping mechanisms and self-efficacy to overcome personal barriers, including stress, which may impact on successful behaviour change [4].

We identified Bandura’s Social Cognitive Theory (SCT) [30] which has been used successfully to change dietary behaviour in other interventions [31, 32] and is a useful theory to inform potential mediating factors involved in dietary behaviour change towards a MD. The SCT acts at the interpersonal level and focuses on the role of observing and learning from others, and on positive and negative reinforcement of behaviour. The key constructs are perceived self-efficacy, outcome expectations, self-regulation and perceived barriers and enablers to enacting the behaviour. These constructs (particularly self-efficacy and self-regulation) have been shown to predict fruit and vegetable intake [31, 32] and fat intake [32, 33], which are also food components of a MD.

In our high CVD risk population, the Health Belief Model (HBM) [34], was also considered important for initiation and maintenance of MD dietary behaviour change at the intrapersonal level. The HBM proposes that successful behavioural change will depend on the individual’s perceived susceptibility to the problem, the seriousness of the consequence of the problem, the perceived benefits of making the change and the perceived barriers to making the desired dietary changes.

Many peer support interventions described in the literature do not clearly describe the detail of the behavioural strategies used by peers to support behaviour change [35]. A recent systematic review [36] and a meta-analysis [37] indicated that social support, goal setting and self-monitoring strategies in interventions are associated with improved dietary behaviours. Less is known about effective BCTs for maintenance of newly adopted dietary behaviour; although engaging social support, setting and reviewing dietary goals, self-monitoring and using problem-solving techniques are thought to be important strategies for longer-term maintenance [38, 39].

##### BCW Stage 2: Intervention functions most likely to bring about behaviour change towards a MD in the target population

We found that five of the nine listed BCW intervention functions were considered most relevant to the COM-B analysis conducted in *BCW Stage 1*. The five intervention functions were: education (increasing knowledge), persuasion (influencing attitudes and actions), training (imparting skills), modelling (using examples for people to aspire to) and enablement (providing support to overcome barriers) [20], as shown in Table 2. These intervention functions were considered most likely to be effective to elicit MD change in the target high CVD risk population using a peer support approach.

### Stage 3

#### MRC Stage 3: Modelling processes and outcomes

Previous reviews have highlighted several components of peer support interventions that may impact on overall effectiveness [40–42] and are important to consider in the early stage of intervention development. These components include: the level of intervention dose and content (behavioural content, frequency and duration of peer support), acceptability of the intervention (user preference for peer support mode of delivery and type of support provided; willingness to change dietary behaviour), the characteristics of peer supporters (skills, attributes, availability, flexibility and personal experiences), and the social environment in which the peer support takes place (family setting and the wider community) [40–42]. Furthermore, many different peer support approaches are described in interventions, for example, face-to-face group or individual programmes, telephone-based or internet-based peer delivered support or a combination of these approaches, and the effectiveness of each have not been conclusively evaluated [43].

Therefore, the peer support intervention format and content was informed by user preferences that were gathered during focus group discussions with the target population and reported elsewhere [44]. In brief, the target population preferred a group-based mode of peer support delivery, either alone or in combination with face-to-face mentoring or telephone support. Preferences were similar across demographics, including gender and geographical location (urban and rural settings). Meeting face-to-face was considered superior to anonymous ‘distance’ approaches and believed to help build trust, foster empathy and facilitate the sharing of personal experiences. Meeting as a group was perceived to create greater opportunity for social engagement to learn from others’ experiences, and, in this way, help to strengthen individual motivation to change dietary behaviours. In addition, qualitative analyses revealed a number of important factors to shape the context, format and content of a peer support intervention including: context (e.g. preference for a convenient community-based location, and for increased frequency of support initially etc.), format (e.g. preference for non-directive, interactive group sessions, based on discussion and shared experience), and, content (e.g. request for information about MD food components, written materials, recipe ideas, tasting sessions, health measurements etc.) [44].

##### BCW Stage 3: Identifying intervention content and implementation options

The Behaviour Change Technique Taxonomy v1 (BCTTv1) [23] and the diet-specific behaviour taxonomy (CALO-RE) [24], were used to select intervention content in terms of BCTs that would best serve the COM-B analysis and the five selected BCW intervention functions. A total of 12 potential BCTs were identified using the BCTT [23] for inclusion in the peer support intervention, as described in Table 2. Some of the identified BCTs were in agreement with successful dietary behaviour change in previous interventions (goal setting, problem solving, social support, action planning and self-monitoring of behaviour), however, several identified BCTs were specific to supporting individual dietary behaviour change in adults at high CVD risk (instruction and demonstration of the behaviour, information about health consequences, setting graded tasks, verbal persuasion, feedback on behaviour and biofeedback. The CALO-RE taxonomy [24] was used to augment and expand on identified BCTs to ensure a comprehensive evaluation of techniques specific to diet behaviour change. Ten BCTs from the CALO-RE taxonomy were considered important for inclusion in the peer support intervention: provide normative behaviour about others’ behaviour, provide information on when and where to perform the behaviour, plan social support/social change, goal setting (outcome), prompt self-monitoring of behaviour, prompt review of behavioural goals, prompt self-monitoring of behavioural outcome, prompt review of behavioural goals, use of follow-up prompts and relapse prevention/coping planning.

A total of 18 BCTs were selected by the research team (*n* = 4) for inclusion in the intervention as shown in Table 3. Selected BCTs were chosen because they were most likely to address COM-B deficits for desired MD behaviour change, acceptable to the target high CVD risk population and feasible to implement by the research team. Two BCTs were not included (verbal persuasion and feedback on behaviour) as these strategies were not considered compatible with a peer support approach. Hence, *capability* to increase MDS will be addressed by offering instruction and demonstration of the behaviour (through written educational material, video and group discussion). *Opportunity* to increase MDS will be enhanced by engaging peer and group social support to encourage dietary behaviour change. Finally, *motivation* to increase MDS will be facilitated by a cluster of self-regulatory BCT’s including problem-solving, goal setting, action planning, self-monitoring, as well as including biofeedback, and information on health consequences, which were important to the target population.

**Table 3:** Behaviour Change Techniques for peer support intervention to encourage dietary change towards a Mediterranean Diet

COM-B domain	BCT for inclusion in the intervention (*n*=19)	Example of intervention strategy to deliver BCT
Capability	Provide normative behaviour about others’ behaviour Provide instruction on how to perform behaviour	Peer supporters provide information about current MD adherence in Northern European populations Peer supporters provide group members with a booklet and a visual guide (MD food pyramid) to explain the types and proportions of food components in a MD
	Model/demonstrate the behaviour Provide information on when and where to perform the behaviour Set graded tasks	Peer supporters show a short video clip to group members demonstrating preparation and consumption of a MD on a budget Recipe books and written information provide information regarding different meals, and also eating out as well as eating in the home. Increasing adherence to a MD is broken down into smaller tasks within written materials, e.g. food swaps are listed separately for each major MD component
Opportunity	Plan social support/social change Social support (unspecified)	Group members are encouraged to support and contact each other between group sessions Peer supporters and group members provide positive encouragement and support to each-other to adopt new MD behaviours
Motivation	Barrier identification/problem solving	Peer supporters facilitate group discussion to identify barriers/ challenges in achieving personal MD goals and assist members to select the best strategies to overcome these
	Goal setting (behaviour) Goal setting (outcome)	Peer supporters support members to set MD goals at each group session based on the session topic Group members are encouraged within their personal planners to define what they want to achieve by taking part in the peer support groups, e.g. weight loss, reduce CVD risk etc.
	Action planning	Peer supporters support members to set MD goals that are easy to measure, something that can be achieved, small and meaningful (i.e. SMART goals) at each group session
	Provide information on consequences of behaviour in general	Peer supporters show a short video clip to group members demonstrating the health effects of a MD
	Biofeedback	Peer supporters offer individual feedback on blood pressure and weight measurements at each group session
	Prompt self-monitoring of behaviour	Group members are given personal planners to monitor their daily/weekly progress in achieving set MD goals and to allow them to record any barriers/challenges they experience
	Prompt self-monitoring of behavioural outcome	Group members are encouraged to log and monitor their weight, blood pressure etc. in personal planners
	Prompt review of behavioural goals	Each group session will provide an opportunity for general progress review in terms of behaviour
	Use of follow-up prompts	Group sessions decrease in frequency after six months
	Relapse prevention/coping planning	One group session (session nine) is dedicated to maintenance of dietary change and relapse prevention

*COM-B* Capability, Opportunity, Motivation – Behaviour Model, *MD* Mediterranean Diet

We developed a range of resources to facilitate MD behaviour change in the population and to optimise delivery of selected BCTs in the peer support intervention. Written educational materials and practical resources to provide information on the MD and associated health benefits alongside practical support, such as meal plans, shopping lists, recipe books and self-monitoring resources were developed specifically to target barriers to dietary behaviour change towards a MD in the population [28]. Intervention implementation options were selected from user preferences for the format, content and delivery of peer support as outlined under MRC stage 3 above. Hence, a group-based peer support intervention was selected as the best approach to encourage dietary change towards a MD in adults at high CVD risk.

### Description of the resulting theory-based peer support intervention to encourage dietary behaviour change in adults at high CVD risk

The tasks performed in three stages within the MRC and BCW frameworks were integrated to develop a tailored, culturally acceptable and theory-based peer support intervention to encourage dietary behaviour change towards a MD. The overall aim of the developed peer support intervention was to engage group-based social support to increase individual capability, opportunity and motivation to change dietary behaviour and to increase MDS by ≥ 3 points over 6 months (adoption phase) and 12 months (maintenance phase) in adults at high CVD risk.

The resulting intervention, delivered by trained ‘lay’ peers, was group-based and consisted of 11 group sessions delivered over 12 months, with 8 sessions delivered in an initial intensive phase (at baseline, 2 weeks, 4 weeks, 6 weeks, 2 months, 3 months, 4 months and 6 months), followed by 3 sessions during a maintenance phase (at months 8, 10 and 12) as guided by qualitative work with our target population [44]. Each session lasted up to 2 hours and the format and content of sessions were designed to be interactive, non-directive and centred on discussion, with emphasis on shared experience and mutual reciprocity between peer supporters and group members for advice and support to achieve MD behaviour change.

Apart from an introductory meeting at baseline, subsequent group sessions had a similar format and theoretical basis as shown in Table 4. Each session had a dedicated discussion topic and included a brief (10-15 minutes) peer-delivered educational component designed to provide a focus for group discussion, as shown in Table 5. The topics were selected to overcome identified barriers to adopting key MD food components in the target population such as increasing olive oil, nuts, fish and fruit and vegetables.

**Table 4:** Individual group session description and BCTs included in the peer support intervention

Session component	Time allocated (min)	Description of session component	BCT(s) included
1. Welcome and attendance register	5	Peer supporter keeps a record of attendance for each session	-
2. General progress review	10	Group discuss their progress in changing dietary behaviour. Positive encouragement and support between members is encouraged	Social support (unspecified)
3. Topic introduction	25	Peer supporter introduces the group session topic. The group share their experiences in relation to the topic and identify the main challenges they have or are likely to encounter in this area	Social support (unspecified) Provide normative behaviour about other’s behaviour
4. Educational demonstration	15	Peer supporters deliver a short educational demonstration such as a video, tasting session, seasonal recipe ideas or a quiz	Instruction on how, when and where to perform the behaviour Demonstrate the behaviour Information about health consequences in general Set graded tasks
5. Supportive discussion	25	Group discussion centres on problem-solving strategies to overcome identified challenges in meeting SMART MD goals or in relation to session topic	Social support (unspecified) Barrier identification/problem-solving
6. Personal action plan	10	Group members are supported to set new or review existing SMART MD goals	Goal setting (behaviour) Goal setting (outcome) Action planning Prompt self-monitoring of behaviour Prompt review of behavioural goals
7. Health measurements	10	Group members are offered an individual measurement of blood pressure and weight. Peer supporter offers personal feedback on health measurements	Biofeedback Prompt self-monitoring of outcome
8. Key messages	10	Group summarise key ‘take home’ messages for the session	Social support (unspecified)
9. Support between sessions	5	Peer supporters encourage ongoing communication and contact between group members outside the scheduled group sessions	Plan social support/social change
10. Next session	5	Peer supporter provides an outline of the next group session	-

*SCT* Social cognitive Theory, *SSM* Social Support Model, *HBM* Health Belief

**Table 5:** Overview of the peer support group-based intervention content to encourage dietary change to a Mediterranean Diet

Session	Delivered	Session topic	Session objectives	Core elements	Resources delivered by peer supporters
1	FIRST SESSION	CHANGING TO A MEDITERRANEAN DIET	Increase awareness and knowledge of the type and proportions of foods in the MD Enable participants to set personal SMART MD goals and to monitor their progress	MD overview SMART goals Monitoring progress	^*^Mediterranean diet pyramid poster
2	WEEK 2	HEALTH BENEFITS OF A MEDITERRANEAN DIET	Identify individual motivators for changing diet towards MD Learn about the health benefits of the MD and discuss practical strategies to promote dietary change	MD and Health Personal Motivation SMART goals Monitoring progress	“Mediterranean diet health benefits” Video Recipe books, shopping lists and meal plan for the season (1)
3	WEEK 4	CHANGING FAT INTAKE	Increase knowledge and awareness of the types and proportions of beneficial fats in MD and increase self-efficacy to change fat intake	Changing fat intake: Replacing SFA with MUFA Sources of MUFA	Oils and nuts to taste
4	WEEK 6	SHOPPING FOR A MEDITERRANEAN DIET	Improve knowledge of MD foods and promote self-efficacy to shop for MD on a budget	Shopping for a MD on a budget	“Healthy Eating on a budget” Video
5	WEEK 8	ENJOY FRUIT AND VEGETABLES	Increase knowledge and awareness of FV as components of MD and promote self-efficacy to increase FV intake	Sources and portions of FV and legumes	“Fruit and Vegetables” Video Fruit and Vegetable portion size guide
6	MONTH 3	EATING A SEASONAL MEDITERRANEAN DIET	Increase awareness of seasonal MD recipes and review personal MD goals in relation to seasonal recipes	MD for the change in season	Recipe books, shopping lists and meal plan for the season (2) Seasonal dish to taste
7	MONTH 4	EATING MORE WHOLEGRAIN	Increase knowledge and awareness of wholegrain intake as a component of MD and promote self-efficacy to increase wholegrain intake	Sources and portions of wholegrain	Wholegrain food cards/quiz Wholegrain portion size guide
8	MONTH 6	EATING A SEASONAL MEDITERRANEAN DIET	Increase awareness of seasonal MD recipes and review personal MD goals in relation to seasonal recipes	MD for the change in season	Recipe books, shopping lists and meal plan for the season (3) Seasonal dish to taste
9	MONTH 8	CONTINUING TO EAT THE MEDITERRANEAN WAY	Review progress in achieving MD SMART goals and promote on-going maintenance of MD Promote maintenance of dietary changes	Relapse prevention	--
10	MONTH 10	EATING A SEASONAL MEDITERRANEAN DIET	Increase awareness of seasonal MD recipes and review personal MD goals in relation to seasonal recipes	MD for the change in season	Recipe books, shopping lists and meal plan for the season (4) Seasonal dish to taste
11	MONTH 12	HOW HAVE WE DONE?	Review progress in achieving MD SMART goals and promote on-going maintenance of MD in the future	MD review Review of progress	--

*This resource is used in each group session; *MD* Mediterranean Diet, *SFA* Saturated fatty acid, *MUFA* Monounsaturated fatty acid, *EVOO* Extra Virgin Olive Oil, *RSO* Rapeseed Oil, *FV* fruit and vegetables

Written educational material (MD health and dietary education and MD pyramid) was provided at the beginning and further written resources were provided in staged format throughout the intervention (e.g. tips and suggestions, meal plans and seasonal recipe ideas) to overcome identified barriers and facilitate dietary behaviour change. MD educational resources aimed to create positive dietary beliefs and attitudes in the population by framing key dietary messages provided to group members in a helpful manner i.e. to ‘replace’, ‘substitute’ or ‘increase’ specific food components, rather than to ‘eat less’, ‘reduce’ or ‘decrease’ foods.

Observational learning was incorporated into the intervention and promoted via practical food demonstrations (using video and food tasting sessions) and through group discussion. To support diet behaviour change [36, 37], a personal planner was developed to encourage group members to record SMART dietary goals, self-monitor their progress, record challenges in making dietary changes and record personal clinical measures (anthropometrics, blood pressure). Group members were encouraged to make small, sustainable changes to their eating behaviour. They could share their goals, progress and challenges to attaining dietary goals with the group, who, in turn, were encouraged to help identify potential solutions via problem-solving discussion facilitated by group peer supporters.

A personal weigh-in and blood pressure measurement was made available for all group members at each session with biofeedback offered by peer supporters. At the end of each session, group member’s summarised key ‘take home’ messages from the session and identified unresolved areas requiring further clarification. The research team provided written answers for any outstanding questions to peer supporters for feedback to the group at the start of the next meeting. Peer supporters also encouraged regular contact between group members beyond the scheduled group meetings (via telephone, text messaging and/or face-to-face meetings) to promote social support and group cohesion.

### Logic model for the peer support MD intervention

**Fig. 2:**
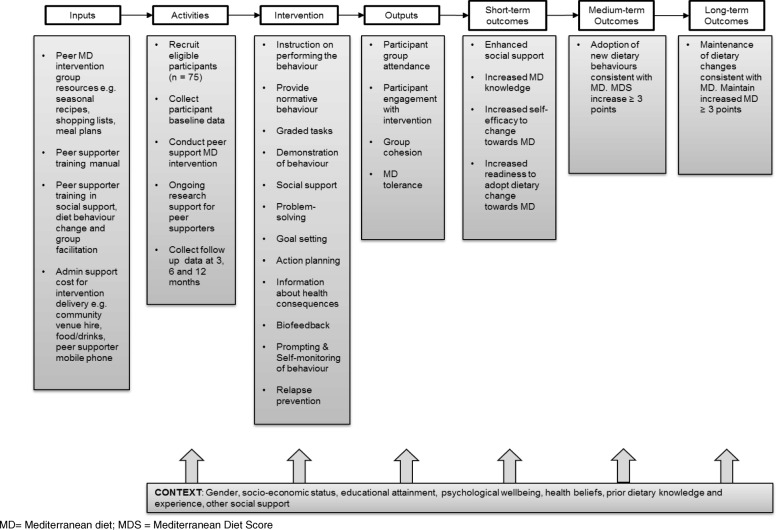
Logic model for the peer support Mediterranean diet intervention

A logic model for the peer support MD intervention is shown in Figure 2 and outlines the intervention inputs, activities, components and proposed outcomes and potential contextual factors which may influence the peer support MD intervention mode of action and outcome measures. This provides a framework for evaluation of the peer support MD intervention to explore the extent to which social-cognitive factors (e.g. knowledge, attitudes, skills, self-efficacy and problem solving in relation to MD), in addition to factors relating to social support, mediate dietary change towards a MD.

## Discussion

This paper describes the process of developing a theory-based, tailored peer support intervention to encourage dietary behaviour change towards a MD in adults at high CVD risk. Both MRC and BCW frameworks were valuable tools used successfully together to guide the intervention development process. The MRC framework was used systematically to identify the evidence base, develop theory and model processes and outcome, while the BCW was used to guide the theoretical basis of the peer support intervention, and tailor the content and format of the peer support intervention to the target population. Using a systematic development process optimises the feasibility of the peer support intervention to encourage MD behaviour change in the target group and minimises the risk of intervention failure. Applying the COM-B model raised our awareness of the significant number of barriers to perceived capability, opportunity and motivation to change dietary behaviour towards a MD in our non-Mediterranean population, but also provided guidance on the most appropriate methods needed to support dietary change. We believe that the behavioural approach to intervention design in the BCW allowed us to make more informed decisions about which active ingredients to include in the intervention and helped us to find the right mix of strategies to drive behaviour change in the population. However, some decisions, particularly regarding which BCTs to include in the intervention, were pragmatic and taken on the basis of feasibility and resources available to deliver the intervention. Therefore, all the theoretical determinants of behaviour that were identified e.g. verbal persuasion and feedback on behaviour, may not be addressed in the developed peer support intervention but will be explored further during evaluation of the intervention. Furthermore, the formative research used to inform intervention development involved a population with relatively low MD adherence, and although we learned much about factors influencing adoption of new behaviours, little information was gleaned about longer-term maintenance of MD behaviour. As mentioned earlier, less is known about BCTs involved in sustained behaviour change, however coping and relapse prevention strategies are considered important. We felt problem-solving was a key BCT to increase motivation and self-efficacy for both adoption and maintenance of MD behaviours and important to include in the peer support intervention. Future planned evaluations of the peer support intervention will aim to identify barriers and enablers to maintain MD behaviour change. We did not perform the ‘policy option’ step recommended in the BCW framework during the intervention development process. This component of the framework will be considered in more detail after the developed peer support intervention has undergone feasibility testing.

Clearly, there are a number of behaviours involved in adopting a MD and the next stage in the process will evaluate ease of adoption across the range of targeted behaviours and the acceptability of changing dietary behaviours in response to the peer support intervention. This approach will allow the intervention to be further adapted and tailored to the needs of the target population. A potential limitation to the peer support intervention is that it is designed to target only dietary behaviour change and does not consider broader lifestyle behaviours besides food consumption, such as physical activity and social interactions that define a Mediterranean lifestyle [45] and are also important modifiable behaviours for CVD prevention [46]

A major strength of the study is that a systematic approach was applied to develop the peer support intervention. Although the process was time intensive taking almost one year to complete, it provides a coherent basis for process evaluation of the intervention. The next stage in the MRC framework recommends pilot testing of the developed intervention. We plan to test the feasibility of the peer support intervention to encourage dietary behaviour change towards a MD in the target population, in comparison to both an intensive support intervention already shown to be effective [2], and a minimal support intervention. The protocol for this pilot RCT is being reported elsewhere and will define randomisation, recruitment strategies, outcome measures and analysis plan.

## Conclusions

In conclusion, the staged MRC and BCW process provided a systematic and complementary approach to developing a theory-based peer support intervention to encourage dietary behaviour change towards a MD in adults at high CVD risk. The next step is to evaluate the feasibility of the peer support intervention to, ultimately, inform the design of a larger scale RCT where the efficacy and cost-effectiveness of the peer support intervention will be tested.

## Abbreviations

BCW: Behaviour Change Wheel
BCTT: Behaviour Change Taxonomy
CALO-RE: Coventry, Aberdeen, and London—Refined taxonomy
COM-B: Capability, Opportunity, Motivation - Behaviour
CVD: Cardiovascular disease
HBM: Health Belief Model
MD: Mediterranean Diet
MDS: Mediterranean Diet Score
MRC: Medical Research Council
RCT: Randomised Controlled Trial
SCT: Social Cognitive Theory
T2DM: Type 2 Diabetes Mellitus
